# High-Throughput Analysis of Ovarian Granulosa Cell Transcriptome

**DOI:** 10.1155/2014/213570

**Published:** 2014-03-10

**Authors:** Ewa Chronowska

**Affiliations:** Third Chair of Surgery, Collegium Medicum, Jagiellonian University, Pradnicka 35-37, 31-202 Cracow, Poland

## Abstract

The quality of follicular oocytes depends on interactions with surrounding granulosa cells. Development of molecular techniques and methods enables better understanding of processes underlying mammalian reproduction on cellular level. The success in reproductive biology and medicine in different species depends on reliable assessment of oocyte and embryo viability which presently mainly bases on embryo morphology. Although successful pregnancies have been achieved using this approach, its precision still should be improved and completed with other, more objective, and accurate assessment strategies. Global profiling of gene expression in follicular cumulus cells using microarrays is continuously leading to the establishment of new biomarkers which can be used to select oocytes with highest developmental potential. Even more potential applications and greater precision could be achieved using next generation sequencing (NGS) of granulosa and cumulus cell RNA (RNA-seq). However, due to the high cost, this method is not used as frequently as microarrays at the moment. In any case, high-throughput technologies offer the possibilities and advantages in ovarian somatic cell analysis on scale that has not been noted so far. The aim of this work is to present current directions and examples of global molecular profiling of granulosa cells and underline its impact on reproductive biology and medicine.

## 1. Introduction

**Figure 1:**
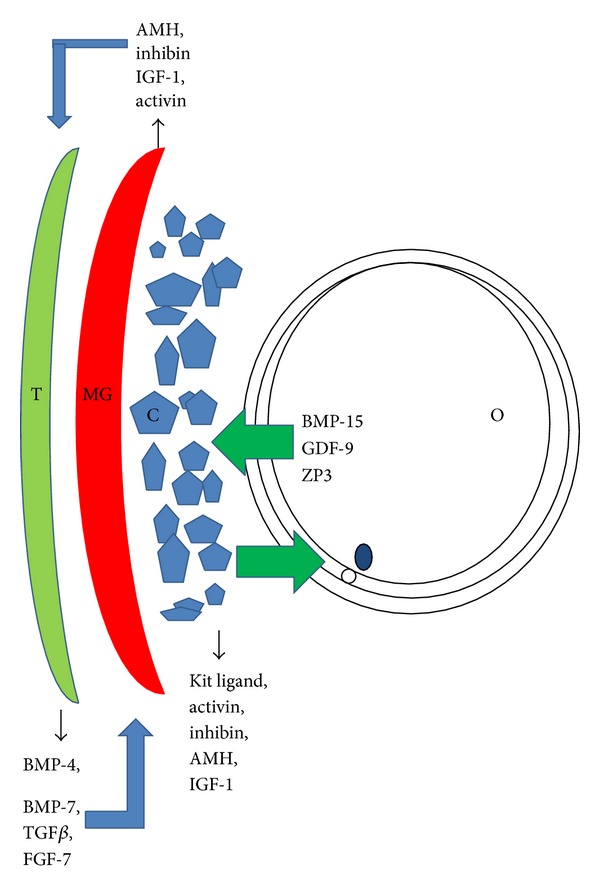
Schematic illustration of mutual interactions (via paracrine growth factors) between somatic cells and oocyte in ovarian follicles (T: theca cell layer, MG: mural granulosa cell layer, C: cumulus cells, and O: oocyte).

Follicles in mammalian ovaries undergo regular changes including recruitment, expansion, maturation, rupture, development into corpus luteum, and final atresia. All those processes are controlled by gonadotropins secreted from the pituitary which administrate signaling networks between the oocyte and the somatic cells in the ovary. Two somatic cell types can be distinguished in the follicle: the mural granulosa cells lying on the basal membrane of the follicular wall and cumulus granulosa cells (CCs) surrounding oocyte. These cells have common origin at early follicular stage but differentiate into two subpopulations in the course of follicular development, up to the preovulatory stage. Cumulus cells stay close to the oocyte forming gap junction projections which allow transport of metabolites between the somatic cells and the oocyte. The oocyte secretes different signaling factors that control and influence proliferation and the differentiation of cumulus cells (Figure 1). Oocyte and cumulus cells form the cumulus-oocyte complex (COC) that remains intact during ovulation up to fertilization. Mural granulosa cell layer remains in less tight contact with oocyte due to the distance and is responsible for the steroidogenic activity of the maturing follicle. Mural granulosa cells express receptors for LH necessary for triggering the final maturation of the follicle and ovulation [1, 2].

The advent and development of “omics” field, such as genomics, transcriptomics, proteomics, and metabolomics, is continuously changing our understanding of mammalian physiology and approaches towards solving particular health problems [3]. In addition to studies aiming at better understanding of basis and processes of mammalian folliculogenesis, increasing interest and significance of high-throughput techniques such as microarrays, next generation sequencing, or single cell genomics is also observed in reproductive medicine to evaluate quality of oocytes and embryos [4].

Transcriptome is the RNA content of the cell [3] while transcriptomics relates to study of transcriptomes and their functions. It serves as a tool to analyse a large number of genes in parallel and bases on assumption that genes involved in specific processes are expressed together and genes showing similar expression patterns may be functionally linked and regulated by similar genetic control mechanisms. Patterns of gene expression levels deliver information about functional status of the cell [5].

**Figure 2:**
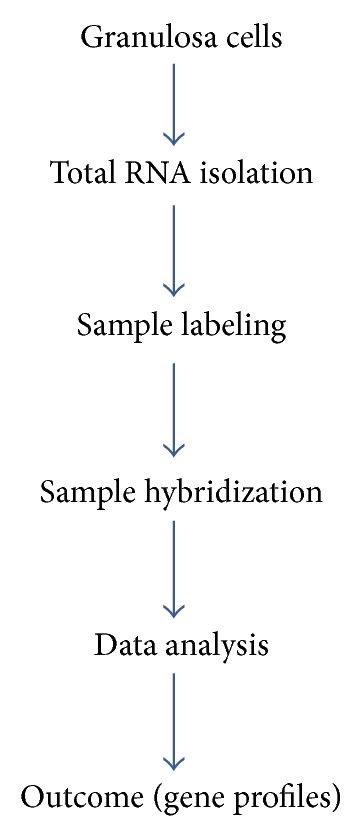
Schematic illustration of preparation of RNA isolated from granulosa cells for transcriptome analysis.

The basic and most widely used approach for transcriptome analysis is microarray hybridization. Currently, several microarray systems are used among which Affymetrix and Illumina platforms are the most popular ones. Illustration of main steps of granulosa cell RNA preparation for microarray analysis is presented in Figure 2.

Next generation sequencing (NGS) is an alternative approach to study qualitative and quantitative RNA content of the cells (RNA-seq). NGS delivers huge amount of data which, after bioinformatic analysis, can be translated into biological knowledge. Although the cost of NGS analysis has been significantly reduced in recent years, it is still considerable which limits application of this technology in routine clinical practice. Nevertheless, this method is being increasingly used in research. It is assumed that NGS, not microarrays, will be the basic tool for genomic and transcriptomic analysis in the future as its potential to deliver biologically relevant information is much greater than in case of approach based on chips.

In this work, current applications, directions, and examples of using high-throughput technologies for investigation of ovarian granulosa cell transcriptome are presented.

## 2. Transcriptomic Analysis of Granulosa Cells to Determine Factors Involved in Follicular Development

Large number of studies have been carried out to identify the mechanisms controlling folliculogenesis. Early folliculogenesis (from follicle formation of the resting pool to the preantral stage) is particularly important as it affects and regulates the size of the resting primordial follicle pool and the fate of the follicles, which in turn influences reproductive life span and fertility [1, 2]. Ovarian follicle is a mixture of different cell types. Cell-specific gene expression driving early folliculogenesis is still not completely known. To elucidate the drive factors of follicular development Bonnet et al. [6] successfully developed laser capture microdissection (LCM) technique coupled with microarray to isolate and investigate very small sized follicles from sheep ovary. Such approach ensured pure cell populations (granulosa cells and oocytes), the transcriptome of which was profiled using bovine chip. The study revealed 1050 transcripts specific to the granulosa cells and 759 specific to the oocyte (comparison of oocyte versus follicular cell transcriptomes). This work, for the first time, demonstrated the global gene expression pattern in specific follicular compartments using high-throughput technology. Obtained data were basis for functional analysis related to processes critical for early folliculogenesis.

To determine crucial factors regulating the gonadotropin-independent and -dependent follicle growth stage and to facilitate development of a culture system for early growing follicles, DNA microarray analysis of mouse ovaries recovered at 7, 10, 13, 16, and 19 days of age was performed [7]. This study showed strong intensity of zona pellucida glycoproteins, bone morphogenic protein-15 (BMP-15), and growth differentiation factor (GDF-9) in 7-day-old mice, which gradually declined in 19-day-old mice. KIT, KIT ligand, anti-Müllerian hormone (AMH), and platelet-derived growth factor (PDGF), which are secreted by granulosa cell secreted factors, also showed relatively high expression. This work contributed to the understanding of factors involved in follicular development and this knowledge may be used for establishment of new in vitro systems for the culture of follicles with potential medical application in severely affected patients.

Using The NimbleGen platform with high-density expression arrays, Batista et al. [8] compared the transcriptomes of granulosa-like cells overexpressing, or not, FOXL2, one of the earliest ovarian markers which, together with its targets, can serve as a model to study ovarian development and function in normal and pathological conditions. It was demonstrated that mediators of inflammation, apoptotic and transcriptional regulators, genes involved in cholesterol metabolism, and genes encoding enzymes, and transcription factors involved in reactive oxygen species detoxification were upregulated. On the other hand, the transcription of genes involved in proteolysis, signal transduction as well as in transcription regulation was shown to be down-regulated by FOXL2. Potential target promoters activated and repressed by FOXL2 were discriminated by bioinformatic analysis.

Using genome-wide analysis of DNA microarray data sets based on samples from periovulatory ovaries, Kawamura et al. [9] found increases in natriuretic peptide precursor type C (NPPC-gene encoding C-type natriuretic peptide, CNP) transcripts in granulosa cells during preovulatory follicle growth in mice and a rapid decline induced by the preovulatory LH/hCG stimulation. Ovarian CNP content was decreased upon treatment of preovulatory animals with hCG. NPPC mRNA was predominantly expressed in mural granulosa cells exhibiting similar regulation following gonadotropin treatment, in isolated ovarian cells.

Bonnet et al. [10] identified the genes differentially expressed in pig granulosa cells in the course of the terminal ovarian follicle growth, to obtain a comprehensive view of these mechanisms. In the first step, specific microarray was developed using cDNAs from suppression subtractive hybridization libraries (345 contigs) obtained by comparison of three follicle size classes: small, medium, and large antral healthy follicles. In the next step, a transcriptomic analysis using cDNA probes from these three follicle classes identified differentially expressed transcripts along the terminal follicular growth and genes predictive of size classes (Table 1). The data analysis allowed identifying gene networks important for terminal follicular development.

In order to elucidate the differentiation status and responsiveness to gonadotropin stimulation in ER*β*-null mice, recently, Binder et al. [11] isolated preovulatory granulosa cells from wild-type and ER*β*-null mice using laser capture microdissection. The aim was to examine the genomic transcriptional response downstream of pregnant mare serum gonadotropin (mimicking FSH) and pregnant mare serum gonadotropin/human chorionic gonadotropin (mimicking LH) stimulation. This approach allowed a comparison of in vivo granulosa cells at the same stage of development from both wild-type and ER*β*-null ovaries. ER*β*-null granulosa cells showed altered expression of genes known to be regulated by FSH (Akap12 and Runx2) as well as not previously reported (Arnt2 and Pou5f1) in wild-type granulosa cells. The analysis also identified more than 300 genes not previously associated with ER*β* in granulosa cells (Table 1).

Small RNAs including microRNAs (miRNAs) are now considered as important regulators of follicular development [20]. Using small RNA sequencing (small RNA-seq) Velthut-Meikas et al. [21] determined the miRNA profile of the two intrafollicular somatic cell types: mural and cumulus granulosa cells isolated from women undergoing controlled ovarian stimulation and in vitro fertilization. In total, 936 annotated and 9 novel miRNAs were identified. Ninety of the annotated miRNAs were differentially expressed between mural granulosa cells and cumulus cells. Bioinformatic analysis revealed that TGF*β*, ErbB signaling, and heparan sulfate biosynthesis were targeted by miRNA in both granulosa cell populations, while extracellular matrix remodeling, Wnt, and neurotrophin signaling pathways were targeted by a miRNA in mural granulosa cells.

## 3. Transcriptomic Analysis of Granulosa Cells to Evaluate Oocyte and Embryo Quality

The fact that cumulus cells are in close communication with oocyte via gap junctions and local paracrine factors suggests that their analysis may give reliable information about the oocyte itself. Cumulus cells can be easily collected without compromising the oocyte, which makes them attractive targets for studies on noninvasive biomarkers of oocyte developmental competence [4].

Gene expression profiling of mural rat granulosa cells with Affymetrix rat whole genome array revealed that the most differentially expressed gene, lysyl oxidase, may be a candidate biomarker of oocyte health and can be used for the selection of good quality oocytes for reproductive biology procedures [13] (Table 1).

Using microarrays Assidi et al. [15] aimed at identifying markers of oocyte competence that are expressed in bovine cumulus cells. Candidate genes expressed in cumulus cells which could be valuable and indirect markers of oocyte competence were hyaluronan synthase 2 (HAS2), inhibin betaA (INHBA), epidermal growth factor receptor (EGFR), gremlin 1 (GREM1), betacellulin (BTC), CD44, tumor necrosis factor-induced protein 6 (TNFAIP6), and prostaglandin-endoperoxide synthase 2 (PTGS2). These biomarkers were proposed to be potential candidates to predict oocyte competence and to select higher-quality embryos for transfer (Table 1). Using bovine model Bettegowda et al. [14] identified differences in RNA transcript abundance in cumulus cells harvested from oocytes of adult versus prepubertal animals (characterized by poor oocyte quality) by microarray analysis. It was revealed that four genes encoding for the lysosomal cysteine proteinases cathepsins B, S, and Z displayed greater transcript abundance in cumulus cells surrounding oocytes harvested from prepubertal animals. Functional analysis indicated the role for cumulus cell cathepsins in compromised oocyte competence and resulted in conclusion that cumulus cell cathepsin transcript abundance may be predictive of oocyte quality (Table 1).

These close relations between oocyte and cumulus cells are of particular significance in assisted reproduction procedure. Single embryo transfer is considered the most appropriate way to reduce the frequency of multiple pregnancies following in vitro fertilisation. However, selecting the embryo for single transfer embryo with the highest chances of pregnancy remains a difficult challenge since morphological and kinetics criteria provide poor prediction of both developmental and implantation ability. The oocyte-cumulus interaction through the expression of specific genes helps the oocyte to acquire its developmental competence. Using microarrays Feuerstein et al. [17] attempted to determine genes related to oocyte developmental competence. Gene expression of oocytecumulus was studied according to the nuclear maturity of the oocyte (immature versus mature oocyte) and to the developmental competence of the oocyte (ability to reach the blastocyst stage after fertilisation). Microarray analysis data delivered 308 differentially expressed genes out of which 8 genes were selected according to oocyte developmental competence for further validation. Three of these 8 selected genes were validated as potential biomarkers (PLIN2, RGS2, and ANG). Finally, RGS2, known as a regulator of G protein signalling, was the only gene among selected candidates biomarkers of oocyte competence which covered many factors of variability (Table 2).

Using human Genome U133 Plus 2.0 microarrays Ouandaogo et al. [22] performed an analysis of the genes expressed in human cumulus cells obtained from patients undergoing intracytoplasmic sperm injection. Cumulus cells samples were isolated from oocyte at germinal vesicle, stage metaphase I, and stage metaphase II. Differentially over-expressed genes between the three cumulus cells categories were identified. The mentioned study demonstrated a specific signature of gene expression in cumulus cells obtained from MII oocyte compared with germinal vesicle and metaphase I. As concluded by the authors gene expression profile, which is specific of MII mature oocyte, may be used as predictor of oocyte quality. Although the study failed to list specific markers of oocyte quality, it anyway underlined the distinct gene signature of individual CC samples isolated from oocytes at different stages of development. To assess such signatures is a first necessary step to qualify the cumulus cell status as competent or incompetent.

As the maturation conditions of human cumulus-oocyte complexes might affect gene expression in both oocyte and cumulus cells Ouandaogo et al. [23] compared the transcriptome profiles of cumulus cells isolated from in vivo and in vitro matured COC at different nuclear maturation stages. In this study microarray technology was used used to analyse the global gene expression to compare the expression profiles of CCs from COC at different nuclear maturation stages following IVM or in vivo maturation. Afterwards, selected genes were validated by qPCR. It was found out that, in CCs isolated after IVM, genes related to cumulus expansion and oocyte maturation, such as EREG, AREG, and PTX3, were downregulated, while cell cycle-related genes were upregulated in comparison with CCs from in vivo matured COC from polycystic ovary syndrome and normal responder patients. Using Affymetrix Gene Chip Mouse Genome 430 2.0 array Kind et al. [24] observed that 1593 genes were differentially expressed, with 811 genes upregulated and 782 genes downregulated in mouse IVM compared with IVV cumulus cells; selected genes were validated by real-time reverse transcription-polymerase chain reaction.

Using DNA microarrays Haouzi et al. [25] studied the LH/hCGR gene expression in cumulus cells surrounding oocytes in patients undergoing controlled ovarian hyperstimulation (COS) before ICSI and related it to other ovarian hyperstimulation quality parameters. The transcriptome analysis of CC indicated a variable expression of LH/hCGR among the patients and intrapatients. LH/hCGR mRNA expression was negatively correlated with serum estradiol level on the day of hCG administration. Eighty-five genes, playing role mainly in steroid metabolism and in the ovulation process (including TNFAIP6), were significantly modulated between cumulus cells from patients with a high and a low LH/hCGR expression. No significant differences in LH/hCGR gene expression profile between COS protocols were observed.

Recently, Assou et al. [19] characterized and compared gene expression profiles in cumulus cells of periovulatory follicles obtained from patients stimulated with HP-hMG or rFSH in a GnRH antagonist cycle and studied their relationship with in vitro embryo development, using Human Genome U133 Plus 2.0 microarrays. Genes upregulated in HP-hMG-treated CCs are involved in lipid metabolism (GM2A) and cell-to-cell interactions (GJA5). On the other hand, genes upregulated in rFSH-treated CCs play role in cell assembly and organization (COL1A1 and COL3A1). It was demonstrated that some genes specific to each gonadotropin treatment (NPY1R and GM2A for HP-hMG; GREM1 and OSBPL6 for rFSH) were associated with day 3 embryo quality and blastocyst grade at day 5, whereas others (STC2 and PTX3) were linked to in vitro embryo quality in both gonadotropin treatments. Embryo and blastocyst quality were assessed daily by the embryologists until 5 days after oocyte retrieval. Top quality 8-cell embryos at day 3 were subjected to blastocyst outcome analysis at day 5 (Table 2).

In study of Hamel et al. [16] hybridization data analysis discriminated 115 genes associated with competent human follicles (leading to a pregnancy). Selected candidates were confirmed by Q-PCR: 3-beta-hydroxysteroid dehydrogenase 1, ferredoxin 1, serine (or cysteine) proteinase inhibitor clade E member 2, cytochrome P450 aromatase, and cell division cycle 42 (Table 2).

Global gene expression analysis performed by Vigone et al. [12] showed that developmentally incompetent and competent CCs share similar transcriptomes, with the exception of 422 genes, 97.6% of which were downregulated in incompetent versus competent CSs.This demonstrated that developmental incompetence or competence of antral oocytes can be predicted using transcript markers expressed by their surrounding CCs (i.e., Has2, Ptx3, Tnfaip6, Ptgs2, and Amh) (Table 1). Using a combined microarray and quantitative reverse-transcription polymerase chain reaction approach Iager et al. [26] found a set of 12 genes predictive of pregnancy outcome based on their expression levels in CCs.

Using microarrays, recently, Papler et al. [27] analyzed surrounding mature oocytes that developed to morulae or blastocysts on day 5 after oocyte retrieval. The analysis revealed 66 differentially expressed genes between cumulus cells of modified natural IVF and controlled ovarian hyperstimulation cycles. Further gene analysis showed that the oxidation-reduction process, glutathione metabolic process, xenobiotic metabolic process, and gene expression were significantly enriched biological processes in modified natural in vitro fertilization cycles. The study of Assou et al. [18] showed that the expression of BCL2L11, PCK1, and NFIB in CCs is significantly correlated with embryo potential and successful pregnancy (Table 2).

## 4. Summary 

Infertility is a civilization disease which affects a significant number of couples of reproductive age. It is also a serious medical and financial challenge which has been approached for years with increasing efficiency and with application of different methods and strategies. The progress in the field of reproductive biology strongly depends on basic research and better understanding of processes regulating follicular development and oocyte maturation. This knowledge can be then translated into clinical practice which enables solving of particular problems of fertility. As presented in this review, transcriptomics is increasingly applied to the gamete and embryo assessment which leads to establishment of biomarkers linked to oocyte and embryo quality. “The omics” offer much more reliable and objective way to assess viability of oocytes and embryos in comparison to conventional morphological evaluation. However, some problems related to the wide application of these methods in clinical practice are still faced. Out of them, duration of analysis and relatively high costs should be mentioned as main obstacles. Therefore, a combination of conventional morphological criteria of evaluation with new, fast, inexpensive, easy-to-use, and noninvasive high-throughput techniques is expected to ensure the progress and efficiency of infertility diagnostics and treatment [3]. Development of high-throughput methods will also be beneficial for solving fertility problems in patients suffering from premature ovarian failure and ovarian impairment due to adjuvant therapy for cancer. It also should be mentioned that not only further achievements in the field of transcriptomics but also proteomics (analysis of cell proteome) will be critical for progress in reproductive biology and medicine.

## Figures and Tables

**Table 1:** Granulosa cell gene expression profiling in non-human species (↑ and ↓ denote up- and downregulated genes, resp.).

Species	Type of cell	Quantity of material analysed	Kind of assay	Gene profiles/outcome	Reference
Mouse	Preovulatory granulosa cells from wild-type and ER*β*-null mice	Captured cells from 2-3 animals of the same genotype were pooled, with at least 3 pools collected per cell type	Affymetrix Mouse Genome 430 2.0 GeneChip arrays	*Tigd3, Ak7, Arnt2, Nid2, Eya4, Mbl2, Ooep, Ptgis, Oog1, Pou5f1, Trim61, *and* Tmem182↑ in PMSG primed ER*β*-null mice granulosa cells versus PMSG primed wild-type mice granulosa cells. Epha5, Ank1, Me2, Reln, Cbs, Cdh2, Ces1d, Mpp7, an Susd4↓ in PMSG primed ER*β*-null mice granulosa cells versus PMSG primed wild-type mice granulosa cells *	Binder et al., 2013 [11]

Mouse	CCs from noncompetent antral oocytes	Total 638 COCs	Illlumina Sentrix arrays	*Has2, Ptx3, Tnfaip6, *and* Ptgs2↓ Amh↑* in noncompetent versus competent oocytes	Vigone et al., 2013 [12]
	CCs from competent antral oocytes	Total 1769 COCs			

Rat	Mural granulosa cells from normal developmental competence oocyte-collected follicles (NDC)	4 samples	Affymetrix GeneChip arrays Rat 230.2.	*Lox, Ngfrap1*↑ *Ggbt2*↓ in NDC versus PDC	Jiang et al., 2010 [13]
	Mural granulosa cells from poor developmental competence oocyte-collected follicles (PDC)	4 samples			

Pig	Small, medium, and large antral follicles	Pooled granulosa cells from follicles of three different size classes. Four samples from small follicles, five samples from medium and large follicles	Microarray	*Hadhb, Psmc2, Gsta1, Ctsl, Hspa8, Mgst1, Erp29, Gart, Cyb5, *and*Cct1↑ *in large follicles versus small and medium follicles *Gsto1, Pkm2, Tuba1b, Calu, Cfl1, Dag1, Tubb5, Eef1a, *and* Rps17↑ *in small follicles versus medium and large follicles	Bonnet et al., 2008 [10]

Bovine	Cumulus cells from adult and prepubertal oocytes	Pools of cumulus cells (*n* = 4) collected from adult and prepuber**t**al animals	Bovine cDNA array (Gene Expression Omnibus platform GPL325)	*Ctsb, Ctss, *and* Ctsz*↑ in cumulus cells collected from oocytes with low developmental competence versus those collected from oocytes with high developmental competence	Bettegowda et al., 2008 [14]

Bovine	COCs from 3 to 8 mm follicles	4 samples	Homemade microarray performed using the VersArray ChipWriter Pro	*Has2, InhbA, Egfr, Grem1, Btc, CD44, Tnfaip6, *and* Ptgs2*, markers of oocyte competence, expressed in CCs	Assidi et al., 2008 [15]

**Table 2:** Gene expression profiling of human granulosa and cumulus cells.

Type of cell	Assay	Gene profiles/outcome	Reference
Granulosa cells from aspirated follicular fluid	Microarray	*FDX1, CYP19A1, CDC42, SERPINE2, *and* 3bHSD1,* positively correlated with oocyte developmental competence	Hamel et al., 2008 [16]
Cumulus cells	Whole Human Genome Oligo Microarray 4x44K (Agilent Technologies)	*RGS2,* related to oocyte developmental competence	Feuerstein et al., 2012 [17]
Cumulus cells	Affymetrix HG-U133 Plus 2.0 array	*BCL2L11, PCK1, *and* NFIB*, significantly correlated with embryo potential	Assou et al., 2008 [18]
Cumulus cells from gonadotropin stimulated patients	Human Genome U133 Plus 2.0 microarrays	*NPY1R, GM2A, GREM1 OSBPL6*, *STC2*, and* PTX3, *correlated with embryo quality	Assou et al., 2013 [19]

## References

[1] Gougeon A (2010). Human ovarian follicular development: from activation of resting follicles to preovulatory maturation. *Annales d’Endocrinologie*.

[2] Palma GA, Argañaraz ME, Barrera AD, Rodler D, Mutto AÁ, Sinowatz F (2012). Biology and biotechnology of follicle development. *The Scientific World Journal*.

[3] Seli E, Robert C, Sirard M-A (2010). Omics in assisted reproduction: possibilities and pitfalls. *Molecular Human Reproduction*.

[4] Assou S, Haouzi D, de Vos J, Hamamah S (2010). Human cumulus cells as biomarkers for embryo and pregnancy outcomes. *Molecular Human Reproduction*.

[5] Nambiar PR, Gupta RR, Misra V (2010). An “Omics” based survey of human colon cancer. *Mutation Research*.

[6] Bonnet A, Bevilacqua C, Benne F (2011). Transcriptome profiling of sheep granulosa cells and oocytes during early follicular development obtained by Laser Capture Microdissection. *BMC Genomics*.

[7] Hasegawa A, Kumamoto K, Mochida N, Komori S, Koyama K (2009). Gene expression profile during ovarian folliculogenesis. *Journal of Reproductive Immunology*.

[8] Batista F, Vaiman D, Dausset J, Fellous M, Veitia RA (2007). Potential targets of FOXL2, a transcription factor involved in craniofacial and follicular development, identified by transcriptomics. *Proceedings of the National Academy of Sciences of the United States of America*.

[9] Kawamura K, Cheng Y, Kawamura N (2011). Pre-ovulatory LH/hCG surge decreases C-type natriuretic peptide secretion by ovarian granulosa cells to promote meiotic resumption of pre-ovulatory oocytes. *Human Reproduction*.

[10] Bonnet A, Lê Cao KA, SanCristobal M (2008). In vivo gene expression in granulosa cells during pig terminal follicular development. *Reproduction*.

[11] Binder AK, Rodriguez KF, Hamilton KJ, Stockton PS, Reed CE, Korach KS (2013). The absence of ER-*β*, results in altered gene expression in ovarian granulosa cells isolated from in vivo preovulatory follicles. *Endocrinology*.

[12] Vigone G, Merico V, Prigione A (2013). Transcriptome based identification of mouse cumulus cell markers that predict the developmental competence of their enclosed antral oocytes. *BMC Genomics*.

[13] Jiang J-Y, Xiong H, Cao M, Xia X, Sirard M-A, Tsang BK (2010). Mural granulosa cell gene expression associated with oocyte developmental competence. *Journal of Ovarian Research*.

[14] Bettegowda A, Patel OV, Lee K-B (2008). Identification of novel bovine cumulus cell molecular markers predictive of oocyte competence: functional and diagnostic implications. *Biology of Reproduction*.

[15] Assidi M, Dufort I, Ali A (2008). Identification of potential markers of oocyte competence expressed in bovine cumulus cells matured with follicle-stimulating hormone and/or phorbol myristate acetate in vitro. *Biology of Reproduction*.

[16] Hamel M, Dufort I, Robert C (2008). Identification of differentially expressed markers in human follicular cells associated with competent oocytes. *Human Reproduction*.

[17] Feuerstein P, Puard V, Chevalier C (2012). Genomic assessment of human cumulus cell marker genes as predictors of oocyte developmental competence: impact of various experimental factors. *PLoS One*.

[18] Assou S, Haouzi D, Mahmoud K (2008). A non-invasive test for assessing embryo potential by gene expression profiles of human cumulus cells: a proof of concept study. *Molecular Human Reproduction*.

[19] Assou S, Haouzi D, Dechaud H, Gala A, Ferrières A, Hamamah S (2013). Comparative gene expression profiling in human cumulus cells according to ovarian gonadotropin treatments. *BioMed Research International*.

[20] Schauer SN, Sontakke SD, Watson ED, Esteves CL, Donadeu FX (2013). Involvement of miRNAs in equine follicle development. *Reproduction*.

[21] Velthut-Meikas A, Simm J, Tuuri T, Tapanainen JS, Metsis M, Salumets A (2013). Research resource: small RNA-seq of human granulosa cells reveals miRNAs in FSHR and aromatase genes. *Molecular Endocrinology*.

[22] Ouandaogo ZG, Haouzi D, Assou S (2011). Human cumulus cells molecular signature in relation to oocyte nuclear maturity stage. *PLoS ONE*.

[23] Ouandaogo ZG, Frydman N, Hesters L (2012). Differences in transcriptomic profiles of human cumulus cells isolated from oocytes at GV, MI and MII stages after in vivo and in vitro oocyte maturation. *Human Reproduction*.

[24] Kind KL, Banwell KM, Gebhardt KM (2013). Microarray analysis of mRNA from cumulus cells following in vivo or in vitro maturation of mouse cumulus-oocyte complexes. *Reproduction, Fertility and Development*.

[25] Haouzi D, Assou S, Mahmoud K (2009). LH/hCGR gene expression in human cumulus cells is linked to the expression of the extracellular matrix modifying gene TNFAIP6 and to serum estradiol levels on day of hCG administration. *Human Reproduction*.

[26] Iager AE, Kocabas AM, Otu HH (2013). Identification of a novel gene set in human cumulus cells predictive of an oocyte’s pregnancy potential. *Fertility and Sterility*.

[27] Papler TB, Bokal EV, Tacer KF, Juvan P, Klun IV, Devjak R (2013). Differences in cumulus cells gene expression between modified natural and stimulated in vitro fertilization cycles. *Journal of Assisted Reproduction and Genetics*.

